# A Novel Routing Algorithm for the Acceleration of Flow Scheduling in Time-Sensitive Networks

**DOI:** 10.3390/s20216400

**Published:** 2020-11-09

**Authors:** Jheng-Yu Huang, Ming-Hung Hsu, Chung-An Shen

**Affiliations:** 1 Department of Electronic and Computer Engineering, National Taiwan University of Science and Technology, Taipei 106, Taiwan; m10602117@mail.ntust.edu.tw; 2 Information and Communications Research Laboratories, Industrial Technology Research Institute, Hsinchu 310, Taiwan; mhhsu@itri.org.tw

**Keywords:** IEEE 802.1Qbv, time-sensitive networking, time-triggered flow, scheduling, routing, computation time, schedulability

## Abstract

IEEE Time-Sensitive Networking (TSN) Task Group specifies a series of standards such as 802.1Qbv for enhancing the management of time-critical flows in real-time networks. Under the IEEE 802.1Qbv standard, the scheduling algorithm is employed to determine the time when a specific gate in the network entities is opened or closed so that the real-time requirements for the flows are guaranteed. The computation time of this scheduling algorithm is critical for the system where dynamic network configurations and settings are required. In addition, the network routing where the paths of the flows are determined has a significant impact on the computation time of the network scheduling. This paper presents a novel scheduling-aware routing algorithm to minimize the computation time of the scheduling algorithm in network management. The proposed routing algorithm determines the path for each time-triggered flow by including the consideration of the period of the flow. This decreases the occurrence of path-conflict during the stage of network scheduling. The detailed outline of the proposed algorithm is presented in this paper. The experimental results show that the proposed routing algorithm reduces the computation time of network scheduling by up to 30% and improves the schedulability of time-triggered flows is the network.

## 1. Introduction

In industrial environments, time-critical tasks such as real-time monitoring and periodic flows of control applications need to be transmitted, processed and responded in a time-guaranteed manner [1,2]. From the perspective of network transmissions, the delay of these time-critical flows needs to be confined within a pre-determined bound; otherwise, catastrophic consequences such as the loss of important messages or the damages of production facilities could take place [3,4]. As a result, proprietary extensions of ethernet have been studied [5,6] for the improvement of real-time responses in the network transmission. In addition, the IEEE Time-Sensitive Networking (TSN) task group strives for the standardized enhancements of the deterministic real-time networks [6,7]. For example, the IEEE 802.1Qbv standard specifies the enhancements of real-time responses for the time-triggered flows [8,9]. A novel traffic shaper, known as the time-aware shaper (TAS), is proposed in the 802.1Qbv standard so that the frames of the time-triggered flows are transported with an accurate clock-synchronized mechanism like IEEE 1588 and IEEE 802.1. To realize the accurately synchronized transmission, time-controlled gates are implemented in the transport ports of the switches or routers for the IEEE 802.1Qbv standard [8,9]. These time-controlled gates need be opened or closed at a scheduled time that is precisely synchronized with the clock. The flow is transmitted when the corresponding gate is opened and is stored in the egress queue waiting to be served when the gate is closed. In such circumstances, a scheduling algorithm is essential to calculate the timing when any specific gate should be opened or closed. The design of this scheduling algorithm has gained significant attention and interest in literature [9,10,11,12,13].

Considering the design of the scheduling algorithm for IEEE 802.1Qbv, it is important to achieve fine-grained scheduling [9] so that the flows are transmitted in a timely manner. On the other hand, the computation time of the scheduling algorithm is also critical especially for the scenarios such as the industry 4.0 concept [14,15,16,17,18] where the dynamic configurations are required and the network settings may change frequently [15,16,17,18,19]. Furthermore, it has been shown in the literature [6,11,13,20] that a scheduling-aware routing scheme which determines the path for each flow while simultaneously considering the scheduling for that flow effectively reduces the computation time of scheduling. In addition, these scheduling-aware routing mechanisms also improve the schedulability, that is, minimize the occurrences where the scheduling algorithm fails to find any acceptable timing schedule. For instance, a routing algorithm based on the K-Shortest Path (KSP) approach [19] is proposed in [6] where the least used routes are selected for the flows. Furthermore, it is shown in [11] that scheduling each time-triggered flow with all available routes improves the schedulability of the scheduling algorithm. Moreover, the work in [13] improves the schedulability by avoiding bottleneck links and striking a balance between the loading of the path and the transmission delay. In addition, the routing algorithm is proposed in [20] aiming to avoid the congestion of the links.

In the context of IEEE 802.1Qbv standard, this paper presents a novel scheduling-aware routing algorithm to reduce the computation time in the network scheduling and improve the schedulability of the time-triggered flows. This algorithm calculates the route for each time-triggered flow by including the consideration of the periods of the flows. Furthermore, the proposed routing algorithm designs the weight for each link edge to improve the schedulability of time-triggered flows. Analytical, as well as experimental results show that the computation time for scheduling is reduced by up to 30% due to the application of the proposed routing algorithm. Moreover, the schedulability of flows is significantly improved compared to the shortest path routing algorithm. The rest of this paper is organized as follows. Section 2 introduces the background and related work. Section 3 introduces the basic concepts and the details of the proposed routing algorithm. Section 4 introduces the experiment results and comparisons with prior arts. Finally, this paper is concluded in Section 5.

## 2. Background and Related Work

The IEEE Time-Sensitive Networking (TSN) 802.1Qbv standard specifies the real-time responses for the time-triggered flows [5,6,7,8,9]. In 802.1Qbv standard the network planning is comprised of two stages, namely routing and scheduling. Specifically, the transmission path of each time-triggered flow is determined in the routing stage by using a certain routing scheme such as the shortest path routing approach [19]. Furthermore, in the subsequent scheduling stage, the scheduling algorithm is performed to decide on the timing schedule for the openness and closeness of the time-controlled gates in the TSN switches along the pre-determined paths [5,9,10]. In addition, for the network planning in 802.1Qbv, the computation time of the scheduling is essential for the scenarios where dynamic configurations are required and the network settings may change frequently [15,16,17,18]. In particular, once the network environment or configuration changes, the scheduling algorithm needs to be executed again to determine the new timing schedules based on the new settings. As a result, an excessive amount of scheduling time results in a delay in the deployment of the network. Even worse, the scheduling algorithm could fail so that no available timing schedules for specified paths can found. This is known as the schedulability issue for the scheduling of the network.

In a typical network planning for the 802.1Qbv, routing and scheduling stages are conducted subsequently and independently. However, it is recently shown [6,11,13,20] that the outcome of the routing stage has an impact on the effectiveness in the scheduling stage, especially for the computational time and schedulability. For example, a routing algorithm based on the K-Shortest Path (KSP) approach [19] is proposed in [6] where the least used routes are selected for the flows. As a result, the delay of flow in the worst-case scenario is decreased and the schedulability of flows is improved. Furthermore, it is shown in [11] that to schedule each time-triggered flow with all available routes improves the schedulability of the scheduling algorithm. Moreover, the work presented in [13] aims to minimize the bottleneck link since it is the cause of the infeasible scheduling of the flow. In addition, the routing of the time-triggered flow with the consideration of load balance as well as the hop counts is employed in [13]. Finally, the algorithm reported in [20] avoids the congestions by considering the load balancing of the flows.

Nevertheless, while aiming for the improvement of the scheduling results, how routing approaches lead to the reduction of the computation time in scheduling has not yet been thoroughly investigated. In this work, we aim to design a novel scheduling-aware routing algorithm so that the computation time of the scheduling is reduced and the schedulability of the time-triggered flows is enhanced in the context of IEEE 802.1Qbv standard.

## 3. The Concept of the Proposed Algorithm

### 3.1. The Hyperperiod and the Scheduling of Time-Critical Flows

When more than one time-critical flows are passing through one edge in the network, the scheduling algorithm must assure that the transmission time for these flows are not overlapped for avoiding the time-conflict on that edge [10]. This can be achieved based on the procedure shown as follows. Firstly, the least common multiple (LCM) for the periods of each flow pair, i.e., the hyperperiod of two flows, on the same edge is computed. Furthermore, the sending time of two different flows are examined to see if there are any conflicts up to the LCM in the time domain on the edge. If no conflict occurs, those two flows will not overlap on that edge in the future since the sending time of these two flows repeats after the LCM. As a result, if two flows of each flow pair do not overlap on the edge up to each LCM, the scheduling for that edge is declared as conflict-free and is feasible.

**Figure 1:**
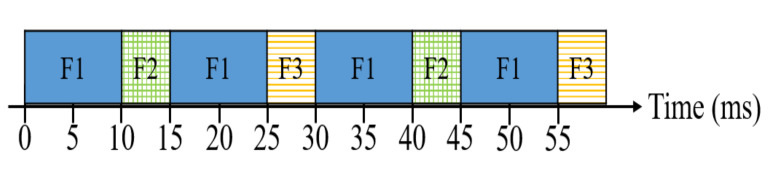
The example of the conflict-free edge.

Figure 1 illustrates an example with three flows where each flow is comprised of one frame. In this example, the periods of flow F1, F2 and F3 are 15, 30 and 30 ms, respectively and the transmission durations of flow F1, F2 and F3 are 10, 5 and 5 ms respectively. For the flow pair of F1 and F2, the hyperperiod is 30 ms, and F1 is sent two times whereas F2 is sent one time before the 30 ms. Therefore, the scheduling algorithm needs to check two times for conflict-free scheduling, i.e., two F1 cannot overlap with F2 until the 30 ms. Similarly, for the flow pair of F1 and F3, the scheduling algorithm needs to check two times for conflict-free scheduling while for the flow pair of F2 and F3, the scheduling algorithm needs to check one time for conflict-free scheduling. According to this example, it can be observed that the hyperperiod of flows on a specific edge has an effect on the number of times for checking if the conflict occurs on the edge. Specifically, it can be seen from this example that the number of times for checking the conflict for one edge increases with the hyperperiods of the flows.

### 3.2. The Concept of the Proposed Routing Scheme

**Figure 2:**
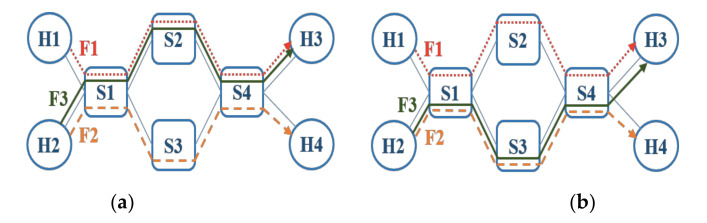
The example of two different routing schemes shown in (**a**) and (**b**) respectively.

It can be seen from Section 3.1 that time-critical flows with higher hyperperiods lead to higher computation times for scheduling as more numbers of checking is needed [10]. Thus, it can be argued that the computation time for scheduling can be decreased if the time-critical flows with small hyperperiods are routed on the same edges as much as possible. This can be illustrated by using an example shown in Figure 2 where a network with 4 switches, 4 hosts, and 3 flows is assumed. Furthermore, the periods of flows F1, F2 and F3 are 20, 30 and 10 ms respectively, whereas the hyperperiod of F1 and F3 is 20 ms and that of F2 and F3 is 30 ms. Moreover, in this example, we assume two routing schemes for the flow F3 shown in Figure 2a,b, respectively, wherein Figure 2a the route for F3 includes edges [H2, S1], [S1, S2], [S2, S4], and [S4, H3] whereas in Figure 2b the route for F3 contains edges [H2, S1], [S1, S3], [S3, S4], and [S4, H3]. The sending time of two different flows are checked up to the LCM on the edge in the time domain for conflict-free scheduling. For the scheme shown in Figure 2a, F1 and F3 on the edge [S1, S2] and the edge [S2, S4] are checked by two times, while for the scheme of Figure 2b, F2 and the F3 on the edge [S1, S3] and the edge [S3, S4] are checked by three times. Therefore, the number of times for the checking and the computation time in scheduling is smaller for the scheme of Figure 2a than that for Figure 2b.

**Figure 3:**
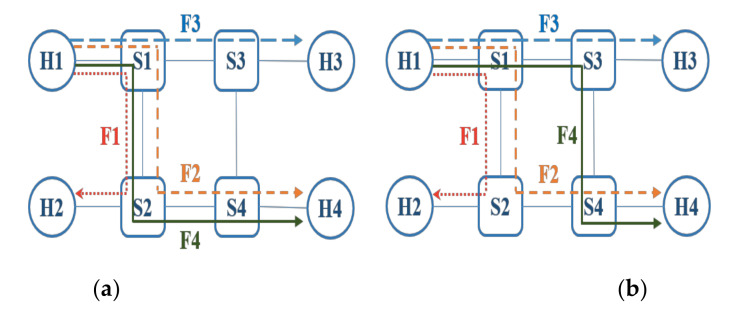
The practical experiment of the example of two different routing schemes shown in (**a**) and (**b**) respectively.

A practical experiment is conducted to highlight the differences in scheduling computation time based on different routing schemes. This experiment is based on a ring topology consisting of 4 switches and 4 hosts as shown in Figure 3. Furthermore, the settings for the experiment are summarized in Table 1 where it can be seen that the hyperperiod of the flow F3 and F4 is smaller than that of F2 and F3. In this experiment, the optimization objective of the scheduling algorithm is to minimize the overall latency for all flows with two different routing schemes assumed in Figure 3a,b, respectively. It is noted that the difference between the two routing schemes lies in the path of flow F4. Our experiment results show that the routing scheme of Figure 3a leads to a computation time of 25.7 s for scheduling whereas that of Figure 3b leads to the computation time of 15.8 s. The reduction of computation time in the scheme of Figure 3b is because the flow F4 passes through the edge [S1, S3] instead of [S1, S2] and results in a reduced occurrence of the overlap. Therefore, these two examples show that routing flows with a small hyperperiod by using the same edges can effectively save the computation time for scheduling.

## 4. The Flow of the Proposed Routing Algorithm

This section presents the detailed procedure of the proposed routing algorithm that is aiming for reducing the computation time for scheduling and improving the schedulability of the flows. Specifically, as can be seen from the exemplary investigations in Section 3.2, the main objective of the proposed routing algorithm is to route the flows with a small hyperperiod by using the same edges as much as possible. The proposed routing algorithm is comprised of two stages, namely sorting and routing. In the sorting stage, the flows are sorted with the periods and the ordered sequence for routing the flow is determined. In the following routing stage, a modified Dijkstra algorithm is proposed to compute the path for each flow according to the order that is determined in the sorting stage. In this section, we first define the notations and metrics used in the algorithm, followed by the detailed flow of the algorithm.

### 4.1. Definition of Notations and Metrics in the Algorithm

In this work, the topology is modeled as directed graph *G*(*N, E*) where the set *N* consists of nodes like switches and end-users and the set *E* consists of edges between the two nodes. Furthermore, the edge between the node *a* and *b* is notated as [$n_{a},\;n_{b}$]. In particular, flows are period unicast data transmissions from a source node to a destination node, and each flow $f_{i}$ is a member of the set of flows *F*. The period of a flow is denoted as $f_{i}.T.$ The hyperperiod of flows $f_{i}$ and $f_{j}$ on the edge [$n_{a},n_{b}$] is noted as $hp_{i}^{j}$ and is computed by the least common multiple (LCM) of periods of the flows as shown in Equation (1).

(1) $$hp_{i}^{j}=lcm\left(f_{i}^{\left[n_{a},n_{b}\right]}.T,\;f_{j}^{\left[n_{a},n_{b}\right]}.T\right)$$

Moreover, parameters α and β are defined to represent the number of times the flows $f_{i}$ and $f_{j}$ pass through the edge [$n_{a},n_{b}$] up to the time of hyperperiod $hp_{i}^{j}$. These two parameters are computed according to each flow pair $(f_{i}^{\left[n_{a},n_{b}\right]},\;f_{j}^{\left[n_{a},n_{b}\right]}$) on each edge [$n_{a},n_{b}$] as expressed in Equation (2).

(2) $$\alpha =\frac{hp_{i}^{j}}{f_{i}^{\left[n_{a},n_{b}\right]}.T},\;\beta =\frac{hp_{i}^{j}}{\;f_{j}^{\left[n_{a},n_{b}\right]}.T},\;\forall \left[n_{a},n_{b}\right]\in E,\forall f_{i}^{\left[n_{a},n_{b}\right]},f_{j}^{\left[n_{a},n_{b}\right]}\in F,i\neq j$$

In addition, an edge weight $e^{\left[n_{a},n_{b}\right]}.weight$ on the edge [$n_{a},n_{b}$] is defined to represent the number of times to examine the scenario if flows do not overlap on the edge up to the hyperperiod on the time domain. Specifically, each flow pair is examined by α×β times on the edge up to the time of hyperperiod for a conflict-free schedule. As a result, the edge weight can be computed by using Equation (3).

(3) $$e^{\left[n_{a},n_{b}\right]}.weight=\left\{\begin{array}{c} 0,\;\mathrm{if}\;\mathrm{there}\;\mathrm{is}\;\mathrm{no}\;\mathrm{flow}\;\mathrm{on}\;e^{\left[n_{a},n_{b}\right]}\\ \frac{\mathrm{LCM}\left(f_{i}^{\left[n_{a},n_{b}\right]}.T,1\right)}{f_{i}^{\left[n_{a},n_{b}\right]}.T}=1,\;\mathrm{if}\;\mathrm{there}\;\mathrm{is}\;a\;\mathrm{flow}\;\mathrm{on}\;e^{\left[n_{a},n_{b}\right]}\\ \sum \alpha \times \beta ,\;\mathrm{if}\;\mathrm{there}\;\mathrm{are}\;\mathrm{multiple}\;\mathrm{flow}\;\mathrm{on}\;e^{\left[n_{a},n_{b}\right]} \end{array}\right.$$

It is shown in Equation (3) that since α and β are determined by each flow pair, the edge weight is equal to the summation of the product of α and β if there are multiple flow pairs on the edge. Furthermore, if there is only one flow on the edge the edge weight is equal to one, which is a benefit for improving the schedulability of flows avoiding bottleneck edges. On the other hand, if the first flow is routed and the weight of each edge along the route contains a non-zero value, the second flow would be routed using edges where the weights are zero.

### 4.2. The Concept of the Proposed Routing Scheme

**Figure 4:**
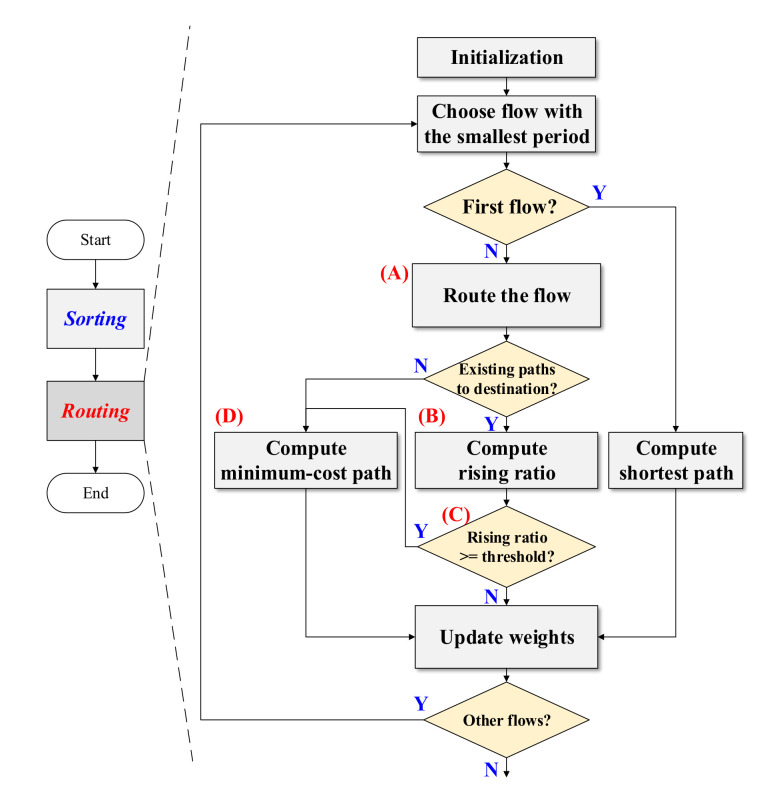
The overall procedure and the procedure for the Routing stage of the proposed algorithm.

Figure 4 presents the overall procedure of the proposed algorithm which is comprised of a Sorting stage and a Routing stage. In particular, the flowchart of the proposed algorithm is shown in Figure 4 and the pseudocode is further provided in Algorithm 1. In the Sorting stage, the flows are sorted with the periods where the flow with the smallest period would be routed first in the Routing stage. The procedure of the Routing stage and the pseudocode of the algorithm are also presented in Figure 4 and Algorithm 1. At the beginning of the Routing stage, the weight of each edge is initialized to zero. In the following, the flow with the smallest period is selected and the shortest path is computed for the selected flow. It is noted that the search area is gradually expanded in the proposed algorithm. In other words, the candidate routes are updated only when another route containing a smaller cost compared to the current route can be found. Furthermore, when routing the first flow, the weight of each available route is zero since the weight of each edge is initialized to be zero. As a result, the first candidate route will not be updated by other routes and the route of the first flow is not only the minimum cost route but also the shortest route. Furthermore, the weight of each edge along the route of the first flow is updated according to Equation (3) and the overall edge weight is updated as the summation of the weight of all edges. The nodes which are visited by the first flow are also updated.
**Algorithm 1**: Proposed Routing Algorithm**Input:***G*(*N*,*E*) and *F* **Output:** Routes 1. **Sort** TT flows with periods. 2. **Initialize** the weight of each edge. 3. **repeat**  4. Choose flow with the smallest period.  5. **if**
*First flow*
**then**    6. Compute the shortest path of the flow.  7. **else**    8. Route the flow with visited nodes, the source node, and the destination node.    9. **if**
*Existing paths to destination*
**then**     10. Compute the rising ratio.     11. **if**
*Rising ratio >= threshold*
**then**      12. Compute the minimum-cost path with all nodes.    13. **else**     14. Compute the minimum-cost path with all nodes.  15. Update weights and visited nodes.  16. **until** There is no unrouted TT flow;

The remaining flows will be routed subsequently after the route for the first flow is determined. It is shown in the flow chart of Figure 4 and the pseudocode of Algorithm 1 that each flow is routed with the visited nodes (i.e., the nodes that have been visited by previous flows), the source node, and the destination node as shown in the block of (A) in Figure 4. In other words, the flow is routed with a part of nodes in the topology and this step attempts to make flows with small hyperperiod pass through the visited nodes. Furthermore, this step results in a scenario where the unvisited nodes can be used for routing the flow which would have a period with prime for the purpose of avoiding a dramatically increased edge weight. As a result, this step of routing flows with visited nodes, the source node, and the destination node leads to a reduced computation time in scheduling. In the following step of block (B) shown in Figure 4, a rising ratio is computed if there is an available route for a flow. This rising ratio is defined in Equation (4) to represent the impact of overall edge weight caused by the flow routed in the iteration.

(4) $$rising\;ratio\;=\;\frac{new\;overall\;edge\;weight\;-old\;overall\;edge\;weight}{old\;overall\;edge\;weight}\times 100\%$$

It can be seen from Equation (4) that a high rising ratio shows that the route of the flow significantly increases overall edge weight. Since the edge weight represents the number of examinations for the scenario if flows do not overlap on the edge up to the hyperperiod time, a high rising ratio caused by the flow also indicates an increased complexity and computation time in scheduling. As a result, the route with a high rising ratio needs to be avoided for reducing the computation time in scheduling.

In addition, in the following step of (C) in Figure 4, a rerouting condition is checked to determine if the flow needs to be rerouted to avoid a route resulting in the dramatic increment in the edge weight. To be specific, the flow will be rerouted if the rising ratio is higher than or equal to a pre-defined threshold that is between 0% and 100%. The proper value of the threshold will be further discussed in Section 5.1. It is noted that according to Equation (3), the edge weight is determined by the summation of the product of *α* and *β* if multiple flows pass through the edge. Furthermore, the values of *α* and *β* are affected by the hyperperiod of flows on the edge. In step (D) shown in Figure 4, the flow is rerouted if there is no available path from the source to the destination via visited nodes or the rising ratio is greater higher or equal to the threshold. In this case, the flow is rerouted using all nodes in the network. Moreover, since nodes that are reserved in step (A) can be used to route the flow with dramatically increased edge weight, the minimum cost path of the flow is computed. The weight of each edge, the overall edge weight, and the visited nodes are updated according to the computed route. In particular, the weight of the edge where multiple flows pass through is computed using the variable *α* and the variable *β*, as shown in Equations (1)–(3). The flow with the smallest period will be routed and finally, this algorithm terminates after all flows are routed.

Based on the pseudocode presented in Algorithm 1, the complexity of the proposed routing algorithm can be evaluated. Specifically, it can be observed from Algorithm 1 that each flow is routed by using the Dijkstra algorithm [21,22] with edge weight in the proposed routing algorithm. Furthermore, if the route though visited nodes are not assigned to a flow or the rising ratio is larger than the threshold, the flow will be rerouted. Considering that all flows need to be routed and certain flows could be rerouted, also with the complexity of the Dijkstra algorithm being O(V^2^), the complexity of the proposed algorithm is O(2*n* × V^2^) where n is the number of flows and V represents the number of nodes.

### 4.3. An Example of the Proposed Algorithm

**Figure 5:**
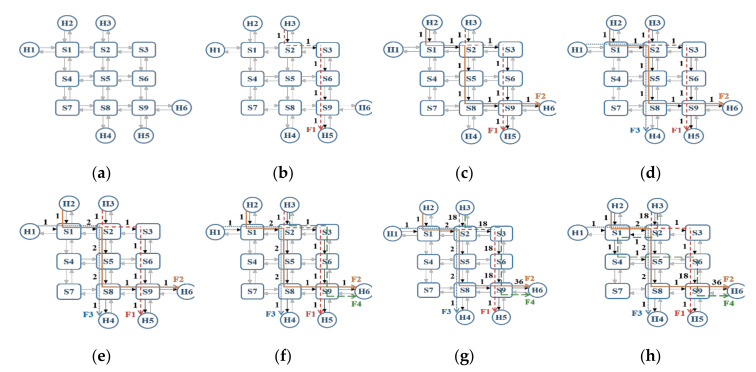
The example that illustrates the operation of the proposed algorithm. (**a**) The mesh topology with 9 switches and 6 hosts. (**b**) The shortest path of the flow F1. (**c**) The path with the minimum cost of the flow F2. (**d**) The result of routing the flow F3 with visited nodes, source node, and the destination node. (**e**) The result of updating the weight of each edge along the path of flow F3. (**f**) The result of routing the flow F4 with visited nodes, the source node, and the destination node. (**g**) The result of computing the weight of each edge where the flow F4 passes through. (**h**) The result of rerouting the flow F4 with all nodes in the topology.

An example is given to illustrate the operation of the proposed algorithm. This example considers four flows in a mesh network containing nine switches, six hosts, and thirty-six edges, whereas the information of each flow is summarized in Table 2. The path of each flow computed by the proposed algorithm is illustrated in Figure 5. To be specific, the initial condition where no flow is routed is shown in Figure 5a. Furthermore, since the order for routing each flow is according to the period of each flow, the flow F1 will be routed first. Thus, the shortest path for F1 is computed and the routing results including the weight of edges, the overall edge weight, and visited nodes along the path are updated as shown in Figure 5b. Moreover, since the F2 cannot be routed with the visited nodes, the source node, and destination node due to that fact that S1 is not visited by any previous flows, the F2 is rerouted with all nodes in the network and the routing results are updated as shown in Figure 5c.

The flow F3 is then routed with the visited nodes, source node, and destination node, as shown in Figure 5d. The rising ratio is then computed by the algorithm which is smaller than the pre-defined threshold (set to be 50% in this example) as shown in Figure 5e. In addition, it is shown in Figure 5f that the flow F4 is routed with the visited nodes, the source node, and the destination node. The rising ratio is computed again and is found to be higher than the threshold, as shown in Figure 5g. Finally, it is shown in Figure 5h that the flow F4 is rerouted with all nodes in the network. It is noted that routing the F4 with all nodes can avoid the overall edge weight increment significantly. This is because the proposed routing algorithm can route F4 with reserved edges. As the weight of each reserved edge is zero, and there is no flow passing through reserved edges, the overall edge weight only increases slightly when routing F4 through reserved edges.

**Figure 6:**
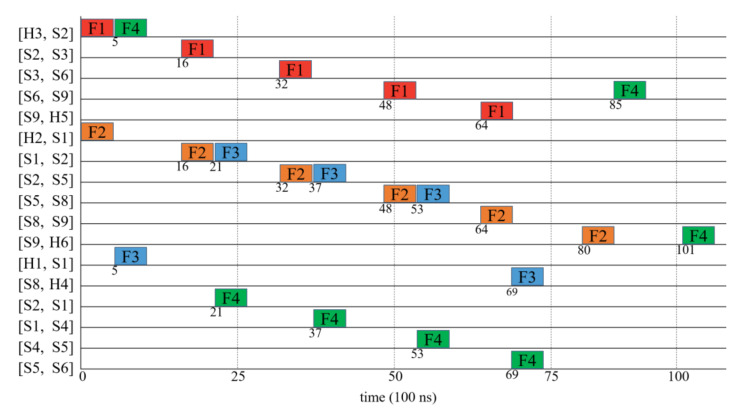
The timelines that flow are transmitted along the routed paths for the example shown in Figure 5.

Figure 6 presents the sending time of each flow on nodes along the path resulted from the proposed routing algorithm. It can be observed from this figure that each flow passes through edges along the path sequentially. For example, the flow F3 passes through edges [H1, S1], [S1, S2], [S2, S5], [S5, S8], and [S8, H4] sequentially. In addition, it can be seen that the proposed routing algorithm attempts to make the F3 pass through visited nodes such as the edges [S1, S2], [S2, S5], [S5, S8] that are also visited by F2. In other words, the F2 is assigned to the unused time slots on the visited edges, and those unrouted flows which would cause the dramatic increase in the edge weight can use reserved edges. For example, the flow F4 is able to be routed with edges [S2, S1], [S1, S4], [S4, S5], [S5, S6].

## 5. Experimental Results and Analyses

This section presents the experimental results and analyses to evaluate the performance and complexity of the proposed algorithm. We first conduct experiments to evaluate the effects of the threshold value that is used in the proposed algorithm. Furthermore, the experiment is conducted to verify the proposed routing algorithm for reducing the computation time in scheduling. A comparison with the seminal shortest path algorithm [22] is also given. Moreover, the experimental results showing that the proposed algorithm improves the schedulability is presented in this section. It is noted that the scheduling algorithm used in our experiments is based on the approach shown in [10] with additional frame constraints which introduce extra limits. These limits request that frames of a frame offset. The flow isolation is also considered in our experiment and the order of the last pair of flow isolation is the same as one of the link constraints. The lower bound of the queue assignment is greater than or equal to zero. All our experiments are conducted based on a 64 bit 4-core 3.40 GHz Intel Core-i7 PC with 16 GB memory in Windows 10 and Z3 v4.6.0-1 [23] is used as the underlying solver.

### 5.1. The Evaluation of the Threshold Values

**Figure 7:**
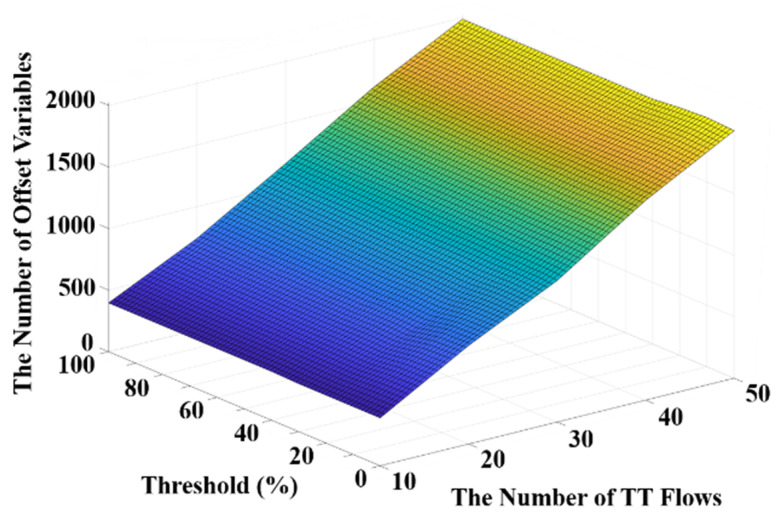
The frame offsets with thresholds and different numbers of flows.

Experiments are conducted to evaluate the impacts of the threshold value to the proposed algorithm from the perspectives of offset variables, the computation time for routing and scheduling, the overall edge weight. Our experiments are based on the network topology Orion [6] consisting of 31 end systems and 15 switches. The size for each flow is configured between 1 and 8 times MTU, whereas the period sets are {10, 20 ms}, {5, 10, 210, 500 ms}, and {2500, 3300, 5000, 6600 µs}. These configurations of flow sizes and period sets are referred to [10]. The source node and the destination node of each flow are determined randomly and the scheduling optimization options are latency minimization, queue usage minimization, and no optimization. Furthermore, as the hyperperiod of flows affects the computation time in scheduling [10], we conduct the threshold experiment with complex period sets to observe the effect of the large hyperperiod on the proper selection of the threshold. Figure 7 presents frame offsets of a flow (i.e., the sending time of frames on each node along the route of the flow) with different thresholds and different numbers of flows. Specifically, a higher offset value indicates a longer route of the flow. It can be observed from Figure 7 that the offset value increases significantly with an increased number of flows, whereas the value of the threshold does not have a significant impact on the offsets.

**Figure 8:**
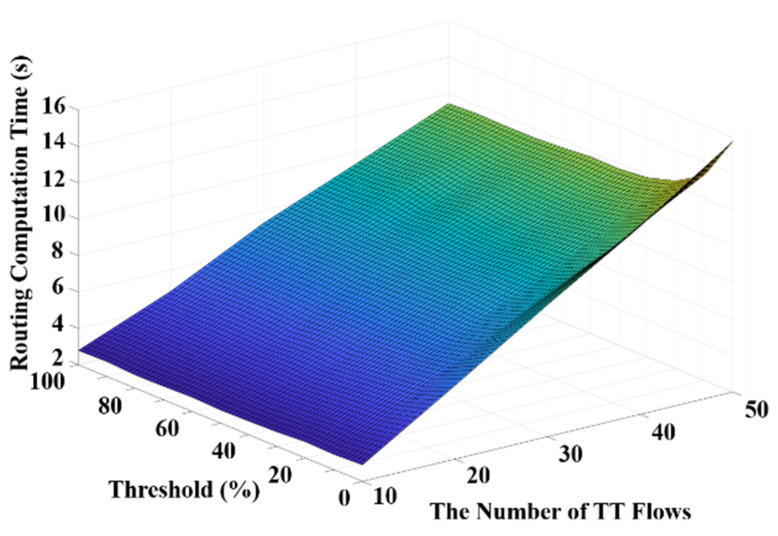
The computation time in routing with thresholds and numbers of flows.

**Figure 9:**
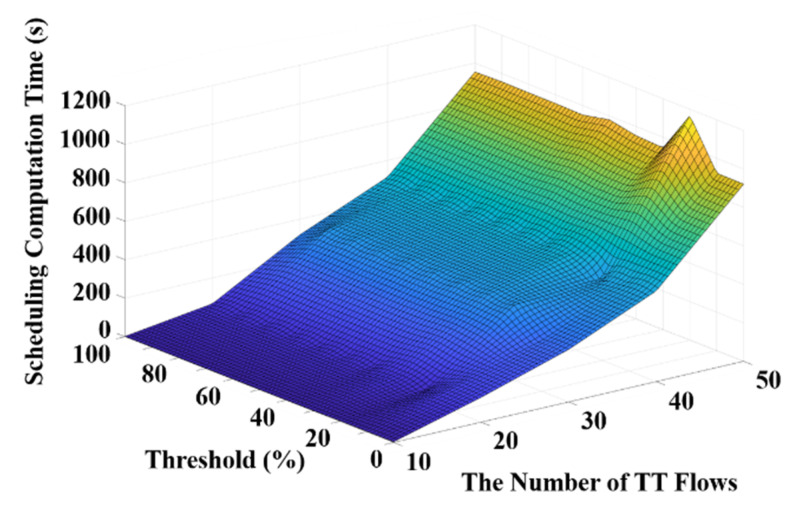
The computation time in scheduling with thresholds and numbers of flows.

**Figure 10:**
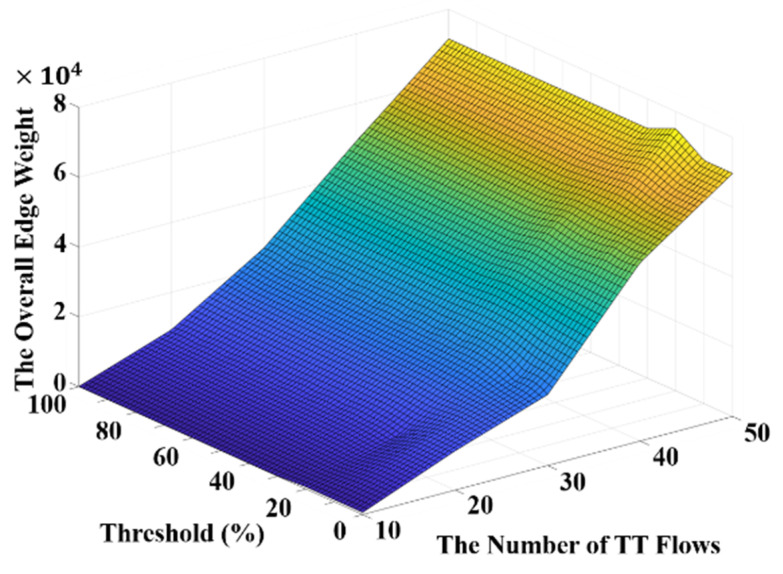
The overall edge weight with thresholds and different numbers of flows.

Furthermore, the average computation time in routing with different thresholds and different numbers of flows is shown in Figure 8. It can be observed in Figure 8 that the computation time of routing drastically increases if the threshold becomes too low. This is because the threshold value basically determines if a flow should be re-routed and thus a lower threshold leads to more re-routing and increases the computation time. On the other hand, it is shown in Figure 8 that the computation time of routing increases along with the number of flows at any given threshold value. Moreover, the average computation time in scheduling with different thresholds and different numbers of flows is presented in Figure 9. It is shown in Figure 9 that the computation time in scheduling is generally lower when the threshold is smaller than 20%. Based on the experimental results illustrated in Figure 8 and Figure 9, a trade-off between the computation time in scheduling and routing can be witnessed. Therefore, considering the overall computation time including both scheduling and routing, the threshold value of 10% could be a proper selection. In addition, the overall edge weights with different thresholds and different numbers of flows are presented in Figure 10. It can be seen that the overall edge weight with the threshold value of 20% is greater than the other threshold values when the number of flows is 50. Therefore, the computation time in scheduling is higher than the others when the threshold is equal to 20% with 50 flows as suggested in Figure 9.

### 5.2. The Evaluation of Computation Time

**Figure 11:**
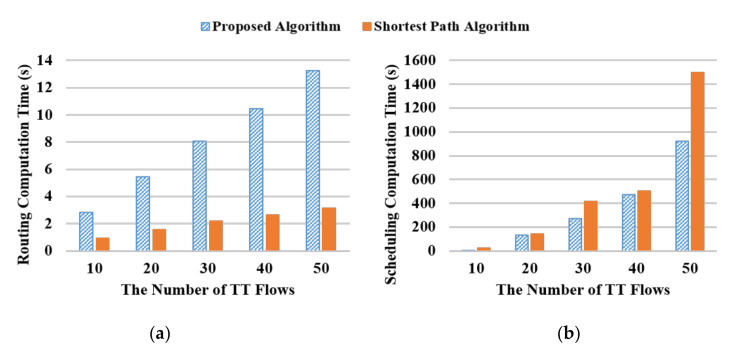
Computation time in (**a**) Routing and (**b**) Scheduling.

The computation time in routing and scheduling with different numbers of flows is investigated. The network topology, period sets, flow size, and scheduling optimization options are the same as the setup for evaluating the thresholds mentioned in Section 5.1. The source node and the destination node of each flow are determined randomly and the threshold is set to be 10%. Figure 11a shows the comparison of computation time between the proposed routing algorithm and the shortest path algorithm (SPA). It can be seen that the computation time for both algorithms increases linearly with the number of flows in the network. Furthermore, the computation of the proposed algorithm is higher than the SPA approach. Moreover, the computation time in scheduling when utilizing the proposed routing algorithm and the SPA routing algorithm is compared in Figure 11b. It can be seen that the proposed routing algorithm results in a much-reduced computation time in scheduling especially when the number of the flow is increased. Specifically, the proposed routing algorithm reduces the computation time in scheduling by 30% on average compared to the SPA scheme.

**Figure 12:**
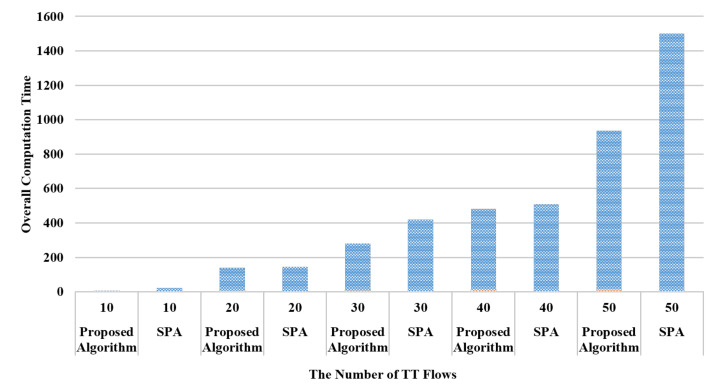
The overall computation time including routing and scheduling.

In addition, it can be observed from Figure 11 that the proposed routing algorithm reduces the computation time in scheduling at the cost of greater computation time in routing. To shed more light on the overall computation time for network planning, it is essential to evaluate the computation time by jointly considering routing and scheduling. Figure 12 compares the computation time in joint routing and scheduling between the proposed routing algorithm and the SPA approach. In other words, the computation time shown in Figure 12 is by adding the computation time shown in Figure 11a with that shown in Figure 11b. It can be observed from this figure that the proposed algorithm can reduce the overall computation time through the tested numbers of flows. Moreover, as the complexity and computation time in scheduling increases significantly with the number of flows, the advantage of the proposed routing algorithm enhances with a greater network.

### 5.3. The Evaluation of Schedulability and Discussion

We also investigate the schedulability of the proposed routing algorithm. In this experiment, the network topology is Orion and Mesh network where each consists of 12 end systems and 14 switches [6] and the threshold is set to be 10%. Furthermore, the period set is {100, 150, 200, 500 µs} and the size of each flow is 1500 bytes. The source node and the destination node of each flow are determined randomly. We evaluate and compare the schedulability between the proposed algorithm and the shortest path routing algorithm (SPA) approach based on the Orion and Mesh network for the number of flows equal to 50, 60, 70, 80, 90, and 100. The results of schedulability experiments are summarized in Table 3. It can be seen from this table that for the Orion network the proposed algorithm is schedulable throughout all tested numbers of flows while the SPA approach cannot be scheduled when the number of flows is larger than 70. In other words, the schedulability for the proposed algorithm is 100% and that for the SPA approach is approximately 33% out of the tested cases for the Orion network. Moreover, it is observed from Table 3 that for the Mesh network the proposed algorithm is schedulable also throughout all tested numbers of flows (i.e., 100% schedulability) while the SPA approach cannot be scheduled when the number of flows is larger than 90 (i.e., approximately 67% schedulability). The experiment shows that the SPA results in the bottleneck edge and leads the unschedulable result. On the contrary, the routes of flows computed by the proposed routing algorithm in both topologies are still schedulable. This is attributed to the fact that the design of edge weight makes flows avoid the bottleneck edge. Therefore, the schedulability can be greatly improved by the proposed routing algorithm.

According to the experimental results shown in Section 5.2 and Section 5.3 that the proposed routing algorithm successfully reduces the computation time in scheduling and improves the schedulability. We believe the effectiveness of the proposed algorithm mainly lies in the fact that the hyperperiod is considered in the routing process. Specifically, it is shown in Figure 1 that the hyperperiod of the flow determines the number of times that to be examined and thus affects the computation time. Thus, the proposed algorithm is designed to avoid a drastic increment of an edge weight measured by the hyperperiod and the number of examinations. As a result, the computation time of scheduling can be minimized. On the other hand, it can be suggested that the proposed algorithm will be less effective for the flows where the scheduling time is inherently short due to the friendlier hyperperiod. Furthermore, the number of disjoint edge path could also affect the performance of the proposed algorithm. In particular, a network topology with less disjoint edge path could degrade the performance of the algorithm since it is more difficult to identify another path. In addition, the flows with the same source and destination nodes are more challenging for the proposed routing algorithm since overlapped edges need to be routed.

## 6. Conclusions

A novel scheduling-aware routing algorithm is presented in this paper aiming to reduce the computation time of network scheduling. The proposed routing algorithm determines the path for each time-triggered flow with the consideration of the period of the flow. The detailed outline of the proposed algorithm is illustrated in this paper and extensive experiments evaluating the performance and computation time of the algorithm are given. The experimental results show that, compared to the conventional routing algorithm, the computation time for network scheduling is reduced by up to 30%, and the schedulability is significantly improved.

## Figures and Tables

**Table 1:** The settings of the flows for the routing examples in Figure 3.

Flow	Source	Destination	Latency (µs)	Size (bits)	Period (µs)
F1	Host 1	Host 2	100	480 × 3	2500
F2	Host 1	Host 4	100	480 × 3	5000
F3	Host 1	Host 3	100	480 × 3	3300

**Table 2:** The information of each flow in the example of Section 4.3.

Flow	Source	Destination	Period (ms)
F1	Host 3	Host 5	2
F2	Host 2	Host 6	4
F3	Host 1	Host 4	8
F4	Host 3	Host 6	9

**Table 3:** Results of schedulability experiments.

	Orion Topology		Mesh Topology	
Flows	Proposed	SPA	Proposed	SPA
50	Yes	Yes	Yes	Yes
60	Yes	Yes	Yes	Yes
70	Yes	No	Yes	Yes
80	Yes	No	Yes	Yes
90	Yes	No	Yes	No
100	Yes	No	Yes	No
